# Enteric Glial Network in Diabetes: Quantitative Changes of Glial Density in Rats in Response to Acute and Chronic Hyperglycaemia

**DOI:** 10.3390/biomedicines14040801

**Published:** 2026-04-01

**Authors:** Benita Onhausz, Bence P. Barta, Abigél Egyed-Kolumbán, Zita Szalai, Mária Bagyánszki, Nikolett Bódi

**Affiliations:** Department of Physiology, Anatomy and Neuroscience, Faculty of Science and Informatics, University of Szeged, Közép Fasor 52, H-6726 Szeged, Hungary; onhausz.benita@bio.u-szeged.hu (B.O.); barta.bence@bio.u-szeged.hu (B.P.B.); egyed.abigel@bio.u-szeged.hu (A.E.-K.); zszalai@bio.u-szeged.hu (Z.S.); bodi.nikolett@bio.u-szeged.hu (N.B.)

**Keywords:** enteric glial cells, Sox10, enteric neurons, acute and chronic hyperglycaemia, type 1 diabetes, insulin, animal model

## Abstract

**Background/Objectives**: Enteric glial cells (EGCs) are key players in regulating enteric neurons and gastrointestinal functions including disturbed gut motility in diabetic patients. Enteric neuronal damage has been shown in type 1 diabetes, but EGCs’ vulnerability to hyperglycaemic insults requires more investigation. Therefore, we aimed to study the quantitative changes in the EGC network enmeshing enteric plexuses, intestinal smooth muscle and mucosa in streptozotocin-induced acute (1-week) and chronic (10-weeks) diabetic rat models. **Methods**: Fluorescent immunohistochemistry using Sox10 glial and HuC/HuD pan-neuronal markers, immunogold electron microscopy and ELISA were performed on different gut segments. **Results**: In the submucosal ganglia of the ileum and colon, the density of Sox10-immunoreactive EGCs was significantly reduced in acute and increased in chronic hyperglycaemic rats without any changes in the duodenum. In the myenteric ganglia, regionally distinct alterations of glial density were noted in acute hyperglycaemia; however, a remarkable decrease was observed in chronic animals. Alterations of neuronal density did not follow the pattern of glial changes, resulting in shifts in the glia/neuron ratio. The presence of Sox10-HuC/HuD-immunoreactive cells and their diabetes-related quantitative changes were also revealed in enteric plexuses. The density of Sox10-labelling gold particles was significantly increased in the duodenal myenteric glia of diabetic rats. Muscular EGC density increased only in the colon after acute hyperglycaemia and changed in all segments after chronic hyperglycaemia. Glial fibrillary acidic protein levels decreased in the small intestine of chronic hyperglycaemic rats. **Conclusions**: Our present findings reveal time-dependent and regionally distinct changes in the EGC network in response to hyperglycaemia, contributing to diabetic enteric neuropathy and gut motility disturbances.

## 1. Introduction

Intestinal motility disturbances accompanying type 1 diabetes can be traced back to complex enteric nervous system damage [1]. The myenteric plexus located between the circular and longitudinal smooth muscle has a crucial role in regulating gut peristalsis, while the submucosal plexus within the submucosa layer of the gut wall is important in glandular secretion, local blood flow and electrolyte transport [2]. While numerous papers listed evidence of neuronal damage in both myenteric and submucosal ganglia under long-lasting hyperglycaemia [3,4,5], more research is needed on the glial components of the gastrointestinal tract in diabetic enteropathy.

Besides being essential for neuronal support, enteric glial cells (EGCs) actively participate in a multitude of gut functions. They regulate mucosal secretion, the intestinal barrier and host defence via interactions with epithelial, enteroendocrine and immune cells [6,7]. EGCs have neurogenic potential [7]: they are able to dedifferentiate and generate enteric neurons in culture [8,9]. Evidence supports EGC involvement in the regulation of gut motility [6] and activation by synaptic neurotransmitters to modulate enteric circuits [6]. McClain et al. [10,11] demonstrated that impairment of glial activity disrupts the neuronal control of peristalsis, leading to constipation in mice. Moreover, EGCs are key players in enteric neuroinflammation: by releasing inflammatory mediators, they create an inflammatory microenvironment for enteric neurons [6,12,13].

The diverse glial roles mentioned above are realized through bidirectional communication between glial and neuronal or non-neuronal cells [14]. Various types of EGCs are present in all layers of the gut wall along the intestinal tract [15]. Different morphological subtypes are located in enteric ganglia, both myenteric and submucosal, interganglionic segments, intestinal smooth muscle and mucosa. Besides morphological differences and physiological properties, diverse and dynamic expression of glial markers, such as glial fibrillary acidic protein (GFAP), S100 and Sox10, also reflects their heterogeneity and phenotypic plasticity [7,16,17,18].

Sox10 is a transcription factor that is expressed in neural crest progenitors and their derivatives and has a crucial role in the development of the enteric nervous system [19,20]. As a special marker of EGCs, its expression level is determinative because it regulates the glial phenotype and function [21]. The role of Sox10 has been demonstrated in central, peripheral and enteric gliogenesis, and it has also been shown that altered Sox10 expressional pattern results in agliogenesis, defects of myelination and intestinal aganglionosis [22].

EGCs are significant players in gastrointestinal disorders such as inflammatory bowel diseases [23]. In these disorders, abnormal glial phenotypes and changes in their number, activity and expression profile were registered [23,24]. Quantitative and morphological alterations of EGCs have also been demonstrated in gastric tissues of diabetic rats, suggesting their role in gastric motility dysfunction in diabetes [25]. Increased reactivity and dysfunction of different central and peripheral glial cells have been observed in both diabetic patients and animal models, highlighting their role in diabetes-related neurodegeneration [26].

Considering that EGCs are active components of all intestinal functions, detailed investigation is essential to evaluate their diabetic damage. Therefore, our main goal was to prepare a gap-filling comprehensive study in which the quantitative features of EGCs were analysed in the myenteric and submucosal ganglia, intestinal smooth muscle and mucosa of different gut segments in acute and chronic hyperglycaemic rat models of type 1 diabetes.

## 2. Materials and Methods

### 2.1. Acute and Chronic Rat Models of Type 1 Diabetes

In this study, adult male Wistar rats (Toxi-Coop Zrt., Balatonfüred, Hungary), kept on standard laboratory chow (Innovo Kft., Zsámbék, Hungary) and drinking water ad libitum, were used.

**Figure 1:**
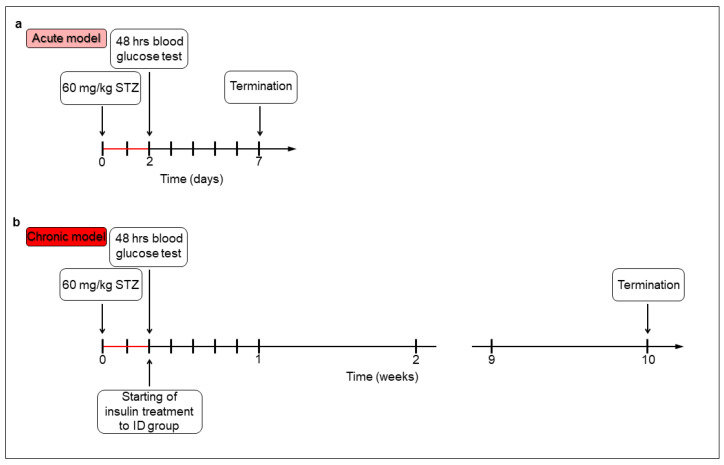
Experimental design of acute (**a**) and chronic (**b**) models of type 1 diabetic rats. Hyperglycaemia was induced by an intraperitoneal streptozotocin (STZ) injection. After 48 h, the non-fasting blood glucose level was determined, and the animals were considered diabetic if it was higher than 18 mmol/L. In the chronic model, a group of hyperglycaemic rats (insulin-treated diabetics, ID) received subcutaneous insulin injections twice a day.

For the acute (1-week) hyperglycaemic experiments (Figure 1a), the animals were divided randomly into 1-week hyperglycaemic (acute hyperglycaemic rats, *n* = 11) and age-matched control (acute controls, *n* = 10) groups. Hyperglycaemia was induced by a single streptozotocin (STZ) injection (i.p. 60 mg/kg, Sigma-Aldrich, Budapest, Hungary) [27,28], while the control group received a vehicle (saline). A total of 48 h later, the non-fasting blood glucose concentration was determined, and the rats were considered diabetic if it was higher than 18 mmol/L [27,28]. Weight and glycaemic parameters of rats were monitored daily under the acute experiment.

For the chronic (10-week) hyperglycaemic experiments (Figure 1b), the rats were divided randomly into 10-week diabetic (chronic diabetic rats; *n* = 12), insulin-treated diabetic (*n* = 12) and sex- and age-matched control (chronic controls; *n* = 11) groups. Induction of hyperglycaemia and criteria were the same as above. The insulin-treated group received insulin injections (Humulin M3, Eli Lilly Nederland, Utrecht, The Netherlands) twice a day (s.c. 3-3 IU). Chronic diabetic and control animals received the same volumes of saline (s.c.). Weights and blood glucose levels were measured weekly. Spontaneously recovered diabetic animals, or whose glucose level decreased under 18 mmol/L were excluded from the study [28].

### 2.2. Tissue Handling

One or ten weeks after the onset of hyperglycaemia, the rats were killed by cervical dislocation under chloral hydrate anaesthesia (375 mg/kg i.p.). Duodenal, ileal and colonic segments of experimental groups were dissected and processed for fluorescent immunohistochemistry, immunogold electron microscopy and ELISA.

For immunofluorescent studies, paraffin-embedded gut sections (5 µm) as well as submucosal and myenteric whole-mounts (fixed in 4% formaldehyde solution overnight at 4 °C) were prepared. Submucosal whole-mount preparations contain the layer of intestinal submucosa, particularly the submucosal plexus, while myenteric whole-mounts include the longitudinal muscle layer with the attached myenteric plexus. For post-embedding electron microscopy, small pieces (2–3 mm) of different gut regions were fixed in 2% paraformaldehyde and 2% glutaraldehyde solution and then further fixed for 1 h in 2% OsO_4_. After rinsing in phosphate buffer and dehydrating in increasing ethanol concentrations and acetone, they were embedded in Embed812 (Electron Microscopy Sciences, Hatfield, PA, USA). Embed blocks were used to prepare 70 nm ultrathin sections. For the ELISA, the 3 cm long gut segments were cut along the mesentery. After removing both the mucosa and submucosa, the intestinal smooth muscle containing the myenteric plexus were snap-frozen in liquid nitrogen and stored at −80 °C until use [28].

### 2.3. Fluorescent Immunohistochemistry

For multi-labelling immunohistochemistry [28], whole-mount preparations and paraffin sections from different gut segments were immunostained with Sox10 and HuC/HuD pan-neuronal marker or Sox10, HuC/HuD and neuronal nitric oxide synthase (nNOS) markers. Briefly, after blocking (1% bovine serum albumin and 10% normal goat serum in TBS), the whole-mounts and sections were incubated overnight with primary antibodies (Table 1) at 4 °C. After washing in TBS with 0.025% Triton X-100, samples were incubated with secondary antibodies (Table 1) for 1 h at room temperature. Negative control samples were made in all cases, where no immunoreactivity was observed. Whole-mounts were mounted on slides in Fluoromount^TM^ Aqueous Mounting Medium (Sigma-Aldrich, Budapest, Hungary), while sections were mounted in Fluoroshield^TM^ with DAPI Mounting Medium (Sigma-Aldrich, Budapest, Hungary) and photographed with a fluorescent microscope (Zeiss Imager Z.2, Axiocam 506 mono camera, Oberkochen, Germany). The number of double-labelled pictures taken from each segment and experimental group of acute and chronic diabetic animals were as follows: 30 to 60 containing submucosal ganglia, 50 to 100 containing myenteric ganglia and 50 of the gut walls showing smooth muscle and mucosa. Cell counting was performed blindly to the treatment groups. The density of Sox10-immunoreactive (IR) glial cells was quantified within enteric ganglia and the intestinal muscle layer and also in the mucosa. The neuronal density and glia/neuron ratio were also determined in enteric ganglia. During quantification, the regions of interest including the area of myenteric and submucosal ganglia were circled, and the number of Sox10-IR glia and HuC/HuD-IR neurons was counted (cells/μm^2^). Triple-labelled pictures were taken from 50 myenteric ganglia of each segment of control animals of the chronic experiment to evaluate the neurochemical code of Sox10-HuC/HuD-IR cells.

### 2.4. Immunogold Electron Microscopy and Morphometry

For the chronic experiment, ultrathin sections were mounted on nickel grids for immunogold labelling [28]. The sections (three grids per block) were incubated overnight in anti-Sox10 (Cat.No.sc-365692, mouse monoclonal, Santa Cruz Biotechnology, Dallas, TX, USA; final dilution 1:50) primary antibody, followed by 18 nm colloidal gold-conjugated anti-mouse IgG (Cat.No.115-215-071, Jackson ImmunoResearch, West Grove, PA, USA; final dilution 1:20) secondary antibody for 3 h. The specificity of the immunoreaction was assessed in all cases by omitting the primary antibody. Sections were counterstained with uranyl acetate (Merck Millipore, Darmstadt, Germany) and lead citrate (Merck Millipore, Darmstadt, Germany) and were photographed with a JEOL JEM 1400 transmission electron microscope (JEOL, Tokyo, Japan). The density of Sox10-labelling gold particles was determined in the nuclei of 20–30 glial cells of 5–10 myenteric ganglia (per segment per condition). The intensity of the labelling was expressed as the total number of gold particles per µm^2^. Digital photographs of myenteric ganglia were also used for ganglionic morphometry to analyse the area of myenteric glial cells (µm^2^) as well as the proportion of neuronal and glial cell bodies’ area per total ganglionic area (Image J 1.54 g).

### 2.5. Measurement of GFAP Concentration of Muscle/Myenteric Plexus Homogenates

Intestinal tissue samples of the chronic experiment, containing the circular and longitudinal smooth muscle with the myenteric plexus in between, were frozen in liquid nitrogen, crushed into powder and homogenised in 500 µL homogenising buffer (100 µL protease inhibitor cocktail (Sigma-Aldrich, Budapest, Hungary) in 20 mL 0.05 M phosphate buffer) and centrifuged (5000 rpm, 20 min, 4 °C) [28]. Quantitative ELISA was used to determine the GFAP levels of the muscle/myenteric plexus homogenates according to the manufacturer’s instructions (GA-E0551RT; GenAsia Biotech Co., Ltd., Shanghai, China). Each sample was measured in duplicate to increase the reliability of the measurements. Optical density was measured at 450 nm (Benchmark Microplate Reader; Bio-Rad, Budapest, Hungary). Tissue GFAP concentrations were expressed as pg/mg protein.

Tissue protein content of gut samples was determined by a commercial protein assay kit. Bradford reagent was added to each sample (10 min), and the samples were assayed spectrophotometrically at 595 nm. Protein level was expressed as mg protein/mL [28].

### 2.6. Statistical Analysis

The Mann–Whitney test (in case of acute experiments) and Kruskal–Wallis test with a Dunn’s multiple comparisons test (in case of chronic experiments) were applied for data analysis by GraphPad Prism 8.0 (GraphPad Software, San Diego, CA, USA). A probability of *p* < 0.05 was set as the level of significance. All data were expressed as the mean ± SEM.

## 3. Results

### 3.1. Body Weight and Blood Glucose Concentration

The body weight and glycaemic parameters of the animals of acute and chronic type 1 diabetic models are represented in Table 2. All rats gained weight during the experiments; however, its extent was smaller in hyperglycaemic groups. Weight gain of control animals was almost twice than it was in the acute hyperglycaemic rats and more than 2.5 times than in the chronic diabetic rats. The blood glucose concentration of hyperglycaemic rats (measured in the mornings) was approximately five times higher than control values (29.85 ± 0.83 vs. 6.52 ± 0.10 mmol/L in acute; 30.04 ± 0.90 vs. 5.99 ± 0.13 mmol/L in chronic). Insulin treatment administered in the chronic experiment prevented extremely high glucose levels (13.44 ± 1.15 mmol/L), but these were still higher than in the control group.

### 3.2. Quantitative Changes in Enteric Glial Cells and Glia/Neuron Ratio in Submucosal Plexus

**Figure 2:**
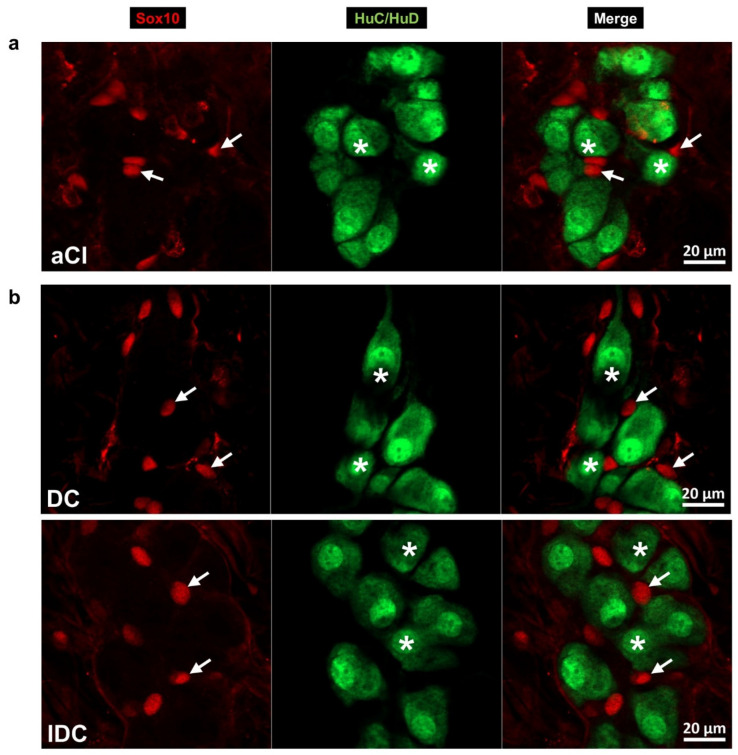
Representative fluorescent micrographs of submucosal ganglia from the ileum of a control rat of the acute experiment (**a**) and the colon of diabetic and insulin-treated diabetic rats of the chronic experiment (**b**) after Sox10-HuC/HuD immunohistochemistry. HuC/HuD as a pan-neuronal marker was used to label enteric neurons. Arrows—Sox10-immunoreactive enteric glial cells; stars—submucosal neurons. aCI—control ileum (acute), DC—diabetic colon, and IDC—insulin-treated diabetic colon.

Double-labelling immunofluorescence with Sox10 glial and HuC/HuD pan-neuronal markers was applied to quantify the enteric glial cells and neurons within the submucosal ganglia (Figure 2).

**Figure 3:**
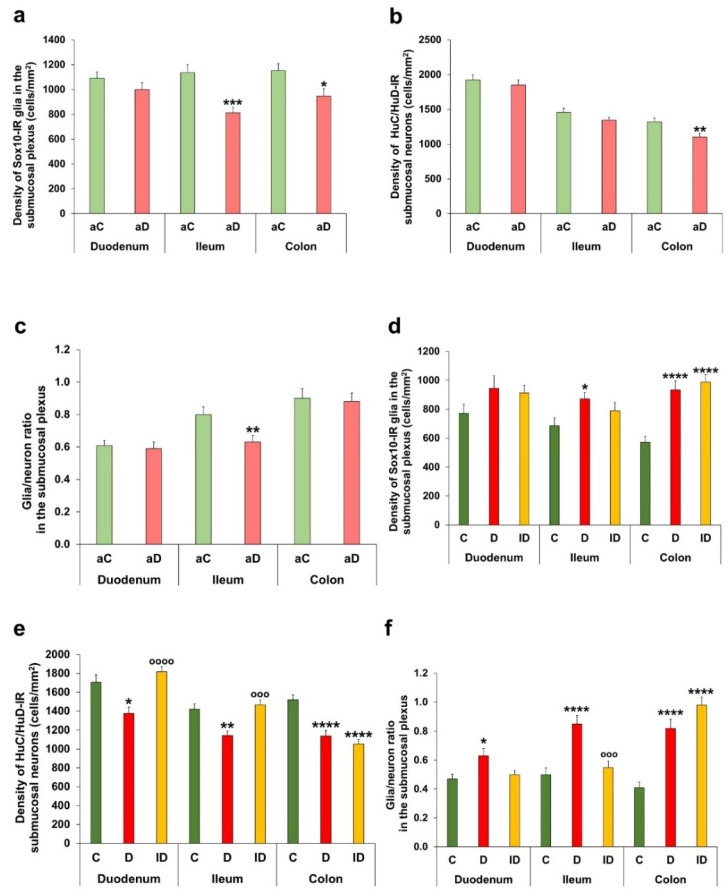
Quantification of enteric glia and neurons in the submucosal plexus. Density of Sox10-immunoreactive (IR) glia (**a**) and HuC/HuD-IR neurons (**b**) and glia/neuron ratio (**c**) in the submucosal ganglia in different gut segments of control and hyperglycaemic rats of the acute experiment. In the submucosal ganglia, acute hyperglycaemia decreased the glial density in the ileum and colon and reduced the neuronal density in the colon. Density of Sox10-IR glia (**d**) and HuC/HuD-IR neurons (**e**) and glia/neuron ratio (**f**) in the submucosal ganglia in different gut segments of control, diabetic and insulin-treated diabetic rats of the chronic experiment. A region-dependent increase in glial density was accompanied with decreased neuronal density in all intestinal segments. This resulted in a definite enhancement of the glia/neuron ratio in the submucosal ganglia of diabetics. Insulin treatment prevented the diabetic changes only in the small intestine. * *p* < 0.05, ** *p* < 0.01, *** *p* < 0.001, and **** *p* < 0.0001 (relative to controls); ^ooo^ *p* < 0.001 and ^oooo^ *p* < 0.0001 (between diabetics and insulin-treated diabetics); Mann–Whitney test ((**a**–**c**) graphs) and Kruskal–Wallis test with Dunn’s multiple comparisons test ((**d**–**f**) graphs). Data are expressed as the mean ± SEM; *n* = 30–60 submucosal ganglia/segment/experimental group from 4 to 5 animals. aC—controls (acute), aD—hyperglycaemics (acute), C—controls (chronic), D—diabetics, and ID—insulin-treated diabetics.

In the acute experiment, the Sox10-IR glial cell number was within a narrow range in all investigated segments of controls. In hyperglycaemic rats, this density significantly decreased in both the ileum (812.86 ± 47.41 vs. 1136.51 ± 63.91 cells/mm^2^ ganglia; *p* < 0.001) and the colon (947.76 ± 58.16 vs. 1151.48 ± 56.88 cells/mm^2^ ganglia; *p* < 0.05); however, it did not change in the duodenum (Figure 3a). In the duodenal submucosal ganglia of hyperglycaemic animals, the density of neurons also remained unchanged (Figure 3b); therefore, the glia/neuron ratio stayed intact here (Figure 3c). In the ileum of hyperglycaemic ratss, the neuronal density was similar to that of controls; therefore, the glia/neuron ratio significantly decreased (*p* < 0.01) following the quantitative changes in glial cells. However, in the colon, the decrease in glial density was accompanied by a decrease in neuronal density (*p* < 0.01), resulting in an unchanged ratio in hyperglycaemic rats compared to controls (Figure 3b,c).

In the chronic experiment, the diabetic density of Sox10-IR glial cells markedly increased in the ileum (871.86 ± 45.12 vs. 685.67 ± 56.84 cells/mm^2^; *p* < 0.05) and the colon (933.70 ± 62.48 vs. 571.25 ± 40.38 cells/mm^2^; *p* < 0.0001) relative to controls, and there was also a slight but not significant increase in glial density in the diabetic duodenum (944.39 ± 86.70 vs. 771.21 ± 64.22 cells/mm^2^) (Figure 3d). Meanwhile, the density of submucosal neurons significantly decreased in the duodenum, ileum and colon of diabetics (Figure 3e), which resulted in the definite enhancement of the glia/neuron ratio in all intestinal segments of the diabetic group (Figure 3f). Immediate insulin treatment completely prevented the diabetic changes in the small intestine; however, it was not able to inhibit the diabetes-related alterations in glial and neuronal densities in the colon (Figure 3d–f).

### 3.3. Quantitative Changes of Enteric Glial Cells and Glia/Neuron Ratio in Myenteric Plexus

**Figure 4:**
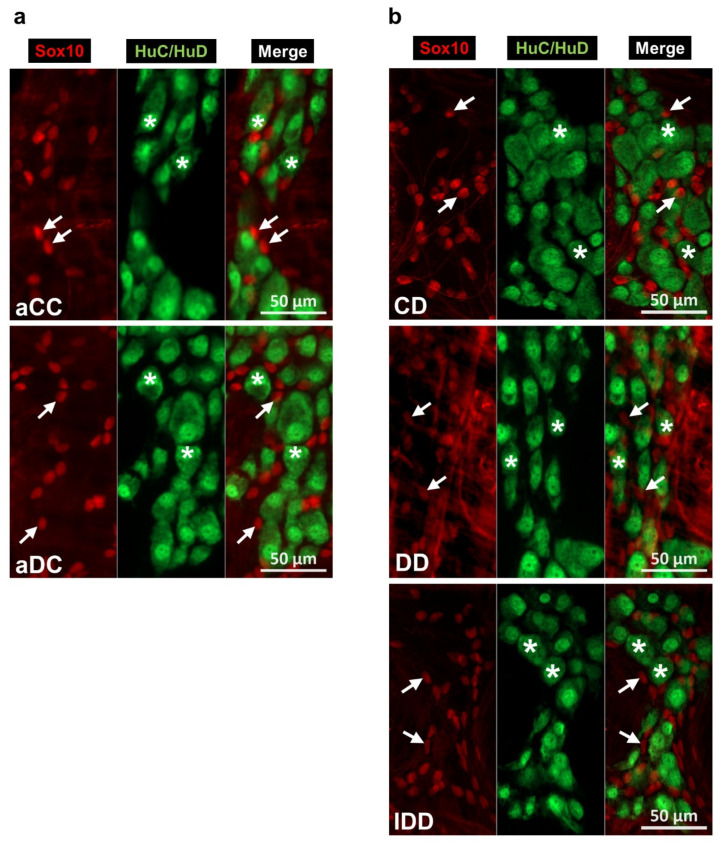
Representative fluorescent micrographs of myenteric ganglia from the colon of control and hyperglycaemic rats of acute experiment (**a**) and the duodenum of control, diabetic and insulin-treated diabetic rats of chronic experiment (**b**) after Sox10-HuC/HuD immunohistochemistry. HuC/HuD as a pan-neuronal marker was used to label enteric neurons. Arrows—Sox10-immunoreactive enteric glial cells; stars—myenteric neurons. aCC—control colon (acute), aDC—hyperglycaemic colon (acute), CD—control duodenum (chronic), DD—diabetic duodenum, and IDD—insulin-treated diabetic duodenum.

Fluorescent immunohistochemistry was used to determine the myenteric glial and neuronal density. Cell bodies and projections of Sox10-IR glial cells and HuC/HuD-IR myenteric neurons completely filled the ganglia (Figure 4).

**Figure 5:**
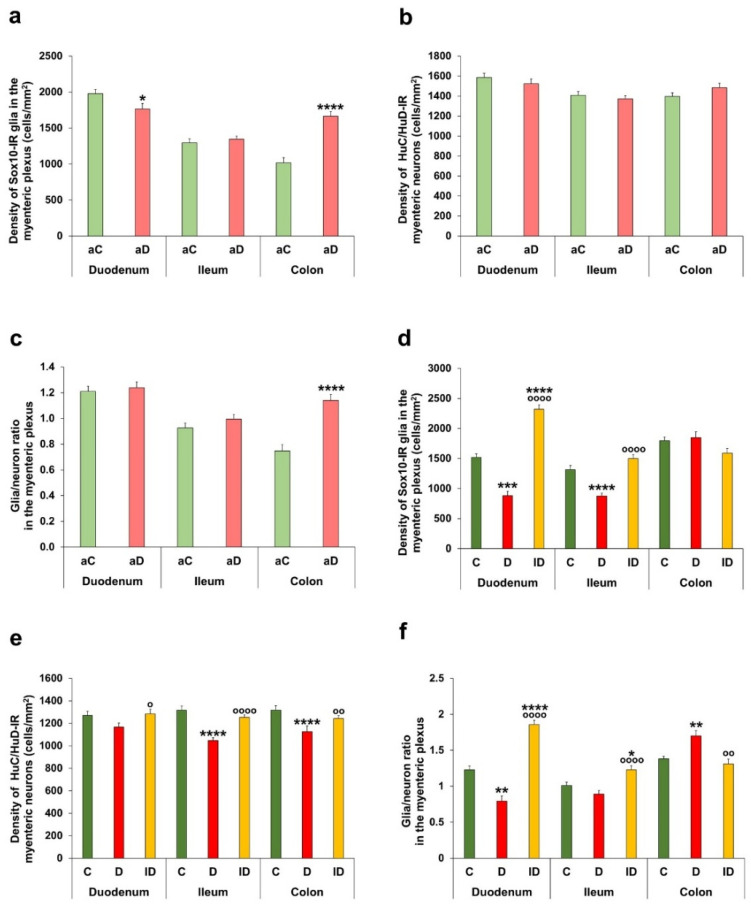
Quantification of enteric glia and neurons in the myenteric plexus. Density of Sox10-immunoreactive (IR) glia (**a**) and HuC/HuD-IR neurons (**b**) and glia/neuron ratio (**c**) in the myenteric ganglia in different gut segments of control and hyperglycaemic rats of the acute experiment. In the myenteric ganglia, acute hyperglycaemia did not affect the neuronal density but caused region-dependent alterations in glial density, leading to a shift in the colonic glia/neuron ratio. Density of Sox10-IR glia (**d**) and HuC/HuD-IR neurons (**e**) and glia/neuron ratio (**f**) in the myenteric ganglia in different gut segments of control, diabetic and insulin-treated diabetic rats of the chronic experiment. Glial density significantly decreased only in the duodenum and ileum, while neuronal density decreased in the ileum and colon of diabetics, which resulted in robust shifts in the glia/neuron ratio after chronic hyperglycaemia. Insulin treatment had segment-dependent preventive effects. * *p* < 0.05, ** *p* < 0.01, *** *p* < 0.001, and **** *p* < 0.0001 (relative to controls); ^o^ *p* < 0.05, ^oo^ *p* < 0.01, and ^oooo^ *p* < 0.0001 (between diabetics and insulin-treated diabetics); Mann–Whitney test ((**a**–**c**) graphs) and Kruskal–Wallis test with Dunn’s multiple comparisons test ((**d**–**f**) graphs). Data are expressed as the mean ± SEM; *n* = 50–100 myenteric ganglia/segment/experimental group from 5 to 7 animals. aC—controls (acute), aD—hyperglycaemics (acute), C—controls (chronic), D—diabetics, and ID—insulin-treated diabetics.

In the myenteric plexus, acute hyperglycaemia did not significantly affect the neuronal density within the ganglia, but it altered the myenteric glial cell density (Figure 5a,b). The glial density was decreased in the duodenum (*p* < 0.05), did not change in the ileum and robustly increased in the colon (1665.56 ± 63.94 vs. 1018.48 ± 68.23 cells/mm^2^ ganglia; *p* < 0.0001) of acute hyperglycaemic animals (Figure 5a). Based on these data, the glia/neuron ratio altered only in the colon; it was increased in acute hyperglycaemia relative to the control state (1.14 ± 0.05 vs. 0.75 ± 0.05; *p* < 0.0001; Figure 5c).

In the diabetic rats of the chronic experiment, a significant decrease was observed in glial density of the duodenum (881.65 ± 73.40 vs. 1518.72 ± 59.53 cells/mm^2^ ganglia; *p* < 0.001) and ileum (877.22 ± 48.56 vs. 1313.67 ± 69.50 cells/mm^2^ ganglia; *p* < 0.0001), while no change was observed in the colon (Figure 5d).

In the duodenum, the diabetic decrease in glial density was not accompanied by neuronal changes; however, in the ileum and colon, neuronal density significantly decreased (*p* < 0.0001; Figure 5e). These resulted in a decreased glia/neuron ratio in the duodenum, unchanged ratio in the ileum and increased ratio in the colon of diabetics (Figure 5f). Insulin treatment prevented the diabetic changes in the ileum and colon and increased the duodenal density of glial cells above control values (*p* < 0.0001) (Figure 5d–f).

### 3.4. Presence and Quantitative Changes of Sox10-HuC/HuD-Immunoreactive Cells in Enteric Plexuses

**Figure 6:**
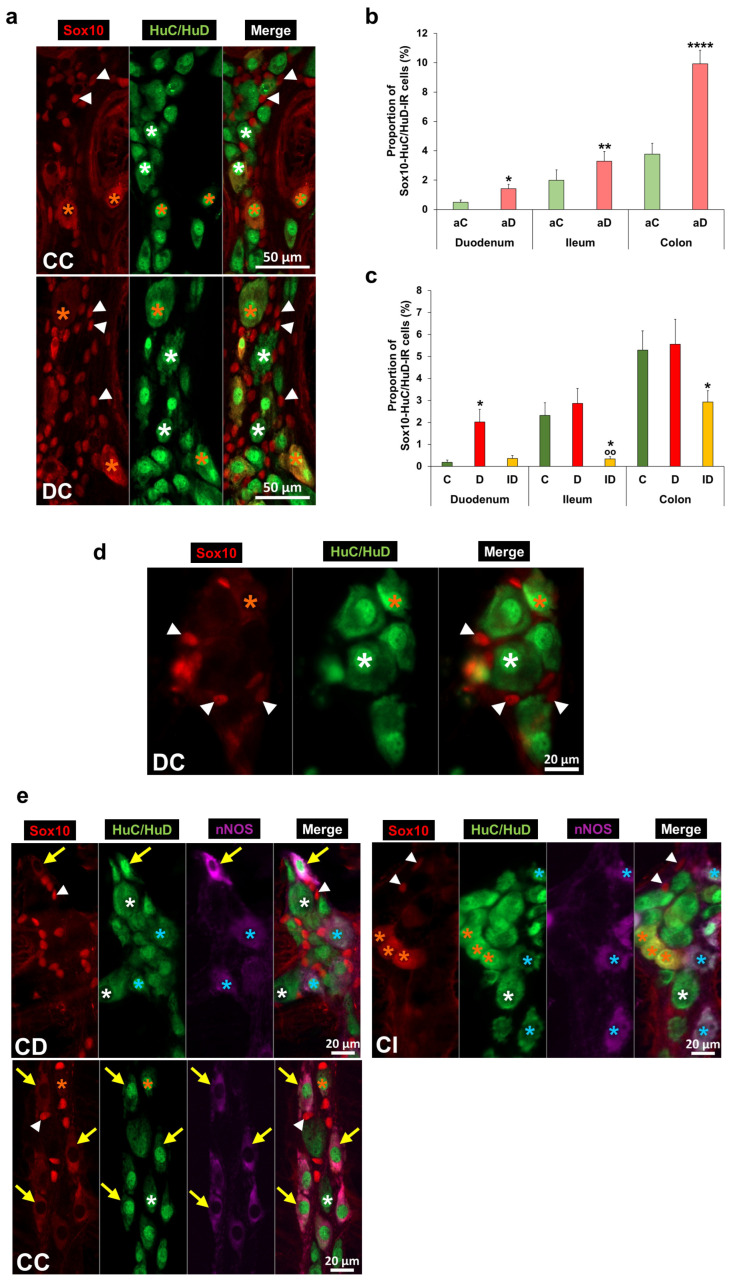
Representative fluorescent micrographs of myenteric (**a**,**e**) and submucosal ganglia (**d**) from the chronic experiment after fluorescent immunohistochemistry. HuC/HuD as a pan-neuronal marker was used to label enteric neurons. Arrowheads—Sox10-immunoreactive (IR) enteric glial cells, white stars—enteric neurons, orange stars—Sox10-HuC/HuD-IR double-labelled cells, blue stars—nNOS-IR myenteric neuron, and yellow arrows—Sox10-HuC/HuD-nNOS-IR triple-labelled cells. CD—control duodenum, CI—control ileum, CC—control colon, and DC—diabetic colon. Proportion of Sox10-HuC/HuD-IR cells in the myenteric ganglia in different gut segments and conditions of the acute (**b**) and chronic (**c**) experiments. The highest proportion of Sox10-HuC/HuD-IR cells was counted in the colon of controls. Acute hyperglycaemia significantly increased that proportion in all gut segments; however, chronic hyperglycaemia resulted in a robust (10-times) increase only in the duodenum. * *p* < 0.05, ** *p* < 0.01, and **** *p* < 0.0001 (relative to controls); ^oo^ *p* < 0.01 (between diabetics and insulin-treated diabetics); Mann–Whitney test (**b**) and Kruskal–Wallis test with Dunn’s multiple comparisons test (**c**). Data are expressed as the mean ± SEM; *n* = 50–100 myenteric ganglia/segment/experimental group from 5 to 7 animals. aC—controls (acute), aD—hyperglycaemics (acute), C—controls (chronic), D—diabetics, and ID—insulin-treated diabetics.

Besides Sox10-IR glia and HuC/HuD-IR neurons, double-labelling immunofluorescence identified Sox10-HuC/HuD-IR cells in the myenteric plexus (Figure 6a). The area of Sox10-HuC/HuD-IR cell bodies did not differ significantly from the area of HuC/HuD-IR neurons (368.4 ± 23.21 µm^2^ vs. 391.57 ± 18.28 µm^2^, *p* = 0.2536).

In the control animals, a high percentage of investigated ganglia contained Sox10-HuC/HuD-IR cells in the colon (acute exp. 65.12%; chronic exp. 70.91%), while it was much lower in the ileum (acute exp. 27.08%; chronic exp. 27.06%) and duodenum (acute exp. 22.41%; chronic exp. 15.56%).

The proportion of Sox10-HuC/HuD-IR cells (per ganglia) was approximately 4–5% in the colon, 2% in the ileum and below 0.5% in the duodenum of controls in both acute and chronic experiments (Figure 6b,c). Acute hyperglycaemia significantly increased that proportion in all investigated gut segments relative to controls (duodenum: 1.42 ± 0.30 vs. 0.48 ± 0.16%, *p* < 0.05; ileum: 3.28 ± 0.68 vs. 1.99 ± 0.72%, *p* < 0.01; colon: 9.92 ± 0.94 vs. 3.78 ± 0.74%, *p* < 0.0001). Chronic hyperglycaemia resulted in regional changes in myenteric ganglia: namely, the proportion of Sox10-HuC/HuD-IR cells displayed a 10-times increase in the duodenum (2.02 ± 0.58 vs. 0.19 ± 0.09%, *p* < 0.05); however, it was not altered in the ileum (2.86 ± 0.68 vs. 2.31 ± 0.58%) and colon (5.55 ± 1.14 vs. 5.29 ± 0.88%) of diabetic rats relative to controls. Insulin treatment prevented the diabetic changes in the duodenum but resulted in the decrease of this proportion below the control values in the other segments (Figure 6b,c).

Triple-labelling immunofluorescence identified that depending on the gut segment, distinct proportions of Sox10-HuC/HuD-IR cells contain nNOS in the myenteric ganglia of control rats (Figure 6e). The proportion of Sox10-HuC/HuD-nNOS-IR cells (per Sox10-HuC/HuD-IR cells) was 90% in the colon and significantly lower in the small intestinal segments of controls (duodenum: 48.26 ± 9.24 vs. 90.28 ± 3.51%, *p* < 0.05; ileum: 18.86 ± 9.42 vs. 90.28 ± 3.51%, *p* < 0.001).

In the submucosal ganglia, we also revealed Sox10-HuC/HuD-IR cells, but they were present in small numbers, which could not be used for quantification (Figure 6d).

### 3.5. Enteric Glial Cells of Mucosa and Intestinal Musculature

**Figure 7:**
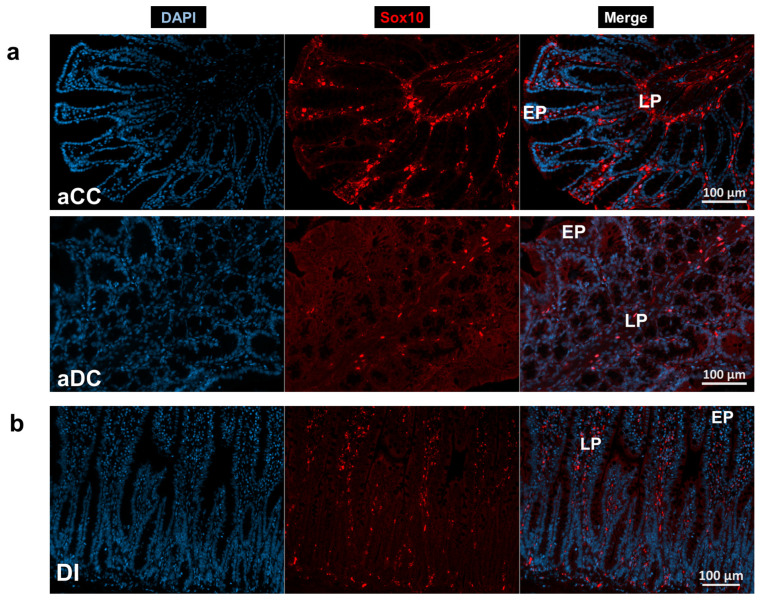
Representative fluorescent micrographs of mucosa from the colon of control and hyperglycaemic rats of the acute experiment (**a**) and the ileum of a diabetic rat of the chronic experiment (**b**) after Sox10 immunohistochemistry. DAPI was applied to label nuclei. aCC—control colon (acute), aDC—hyperglycaemic colon (acute), DI—diabetic ileum, EP—epithelium, and LP—lamina propria.

Besides enteric plexuses, mucosal and muscular EGCs were regionally evaluated in the gut wall by immunofluorescence. In the mucosa, Sox10 immunoreactivity outlined the elements of the EGCs’ network. An extensive mucosal population of EGCs was visible in the lamina propria around intestinal crypts, close to the epithelial cells in all investigated gut segments (Figure 7).

**Figure 8:**
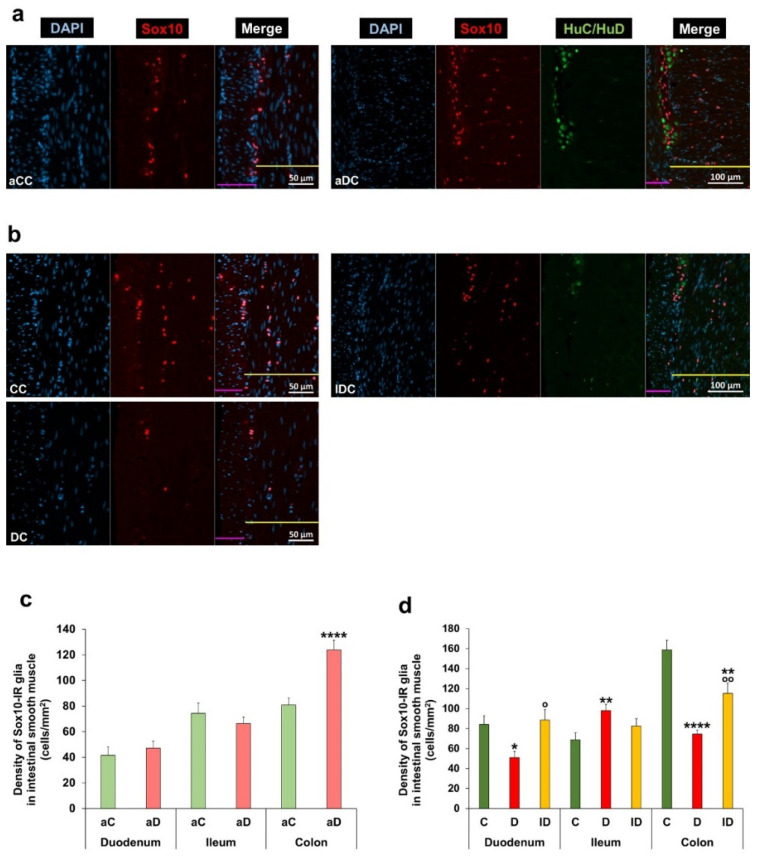
Representative fluorescent micrographs of intestinal smooth muscle layers from the colon of control and hyperglycaemic rats of acute experiment (**a**) and the colon of control, diabetic and insulin-treated rats of chronic experiment (**b**) after Sox10-HuC/HuD immunohistochemistry. HuC/HuD as a pan-neuronal marker was used to label enteric neurons. DAPI was applied to label nuclei. Yellow line—circular smooth muscle; violet line—longitudinal smooth muscle. aCC—control colon (acute), aDC—acute hyperglycaemic colon, CC—control colon (chronic), DC—diabetic colon, and IDC—insulin-treated diabetic colon. Density of Sox10-immunoreactive (IR) glia in the intestinal smooth muscle in different gut segments and conditions of the acute (**c**) and chronic (**d**) experiments. Sox10-IR muscular glial density was increased in the colon of acute hyperglycaemic rats, while it decreased in the duodenum and colon and increased in the ileum of diabetic animals. Insulin treatment completely prevented the diabetic glial changes in the musculature of the small intestine. * *p* < 0.05, ** *p* < 0.01, and **** *p* < 0.0001 (relative to controls); ^o^ *p* < 0.05 and ^oo^ *p* < 0.01 (between diabetics and insulin-treated diabetics); Mann–Whitney test (**c**) and Kruskal–Wallis test with Dunn’s multiple comparisons test (**d**). Data are expressed as the mean ± SEM; *n* = 50 pictures of intestinal smooth muscle/segment/experimental group from 4 to 5 animals. aC—controls (acute), aD—hyperglycaemics (acute), C—controls (chronic), D—diabetics, and ID—insulin-treated diabetics.

Muscular EGCs were located in both the circular and longitudinal layers of intestinal smooth muscle; however, their distribution was unequal. Most Sox10-IR glial cells were observed in the circular muscle layer, while there were only a few cells in the longitudinal layer in all gut segments and conditions (Figure 8a,b). The total density of Sox10-IR muscular glial cells was highest in the colon and lowest in the proximal parts of control animals. In the musculature of acute hyperglycaemic rats, this density was markedly increased in the colon relative to control values (123.87 ± 7.42 vs. 80.88 ± 5.38 cells/mm^2^; *p* < 0.0001), while it remained unchanged in the duodenum and ileum (Figure 8a,c). However, chronic hyperglycaemia had significant effects on the density of muscular glial cells in all investigated gut regions: namely, a robust decrease in the duodenum and colon and increase in the ileum compared to controls (duodenum: 50.92 ± 6.31 vs. 84.23 ± 8.74 cells/mm^2^; *p* < 0.05; ileum: 98.05 ± 6.23 vs. 68.76 ± 7.07 cells/mm^2^; *p* < 0.01; colon: 74.63 ± 3.95 vs. 158.85 ± 9.55 cells/mm^2^; *p* < 0.0001). Insulin treatment prevented the diabetic glial changes in the musculature of the small intestine, but its beneficial effect was only partial in the colon (Figure 8b,d).

### 3.6. Sox10 Density in Myenteric Glial Cells and Ganglionic Morphometry

**Figure 9:**
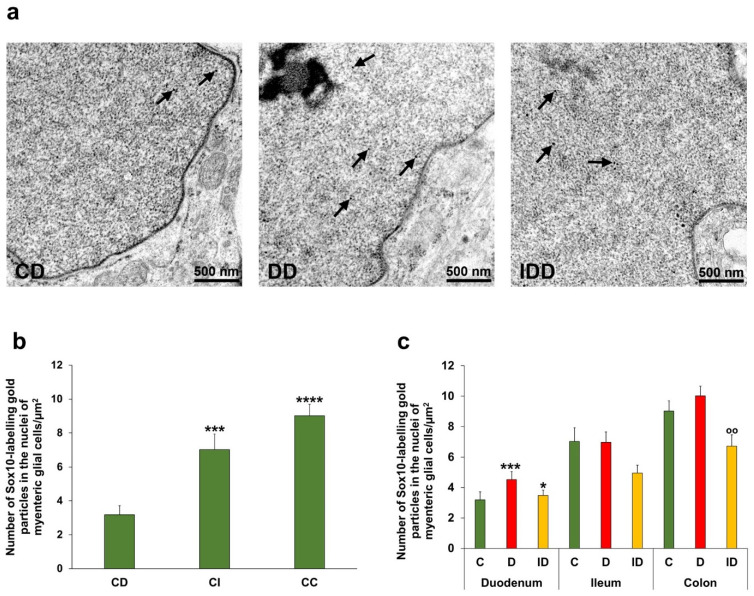
Representative electron micrographs of discrete regions of myenteric glial cells from the duodenum of control, diabetic and insulin-treated diabetic rats of the chronic experiment after Sox10 post-embedding immunohistochemistry (**a**). Arrows—18 nm gold particles labelling Sox10. Quantitative evaluation of gold particles labelling Sox10 in the nuclei of myenteric glial cells from different gut segments of control animals (**b**). Glial Sox10 density was lowest in the duodenum, with a two-fold increase observed in the ileum and a three-fold increase in the colon. *** *p* < 0.001 and **** *p* < 0.0001 (relative to control duodenum). Density of Sox10-labelling gold particles in the nuclei of myenteric glial cells from different gut segments of control, diabetic and insulin-treated diabetic rats (**c**). Sox10 density was significantly increased in the duodenum of diabetic rats, which was prevented by insulin treatment. * *p* < 0.05 and *** *p* < 0.001 (relative to controls); ^oo^ *p* < 0.01 (between diabetics and insulin-treated diabetics); Kruskal–Wallis test with Dunn’s multiple comparisons test. Data were expressed as means ± SEMs; *n* = 20–30 nuclei of glial cells of 5–10 myenteric ganglia/segment/experimental group from 6 to 7 animals. CD—control duodenum, CI—control ileum, CC—control colon, DD—diabetic duodenum, IDD—insulin-treated diabetic duodenum, C—controls, D—diabetics, and ID—insulin-treated diabetics.

Immunogold electron microscopy was used to evaluate the density of Sox10 in myenteric glial cells. Here, 18 nm gold particles labelling Sox10 were accumulated in the glial cells’ nuclei (Figure 9a). Glial Sox10 density was lowest in the duodenum (3.19 ± 0.53 particles/µm^2^), two times higher in the ileum (7.03 ± 0.90 particles/µm^2^; *p* < 0.001) and three times higher in the colon (9.02 ± 0.67 particles/µm^2^; *p* < 0.0001) (Figure 9b). Chronic hyperglycaemia affected the Sox10 density only in the duodenum of diabetic rats, where it was significantly increased (4.53 ± 0.53 vs. 3.19 ± 0.53 particles/µm^2^; *p* < 0.001) (Figure 9c). Insulin treatment prevented the diabetic changes in the duodenum and decreased the Sox10 density in the colon (Figure 9c).

**Figure 10:**
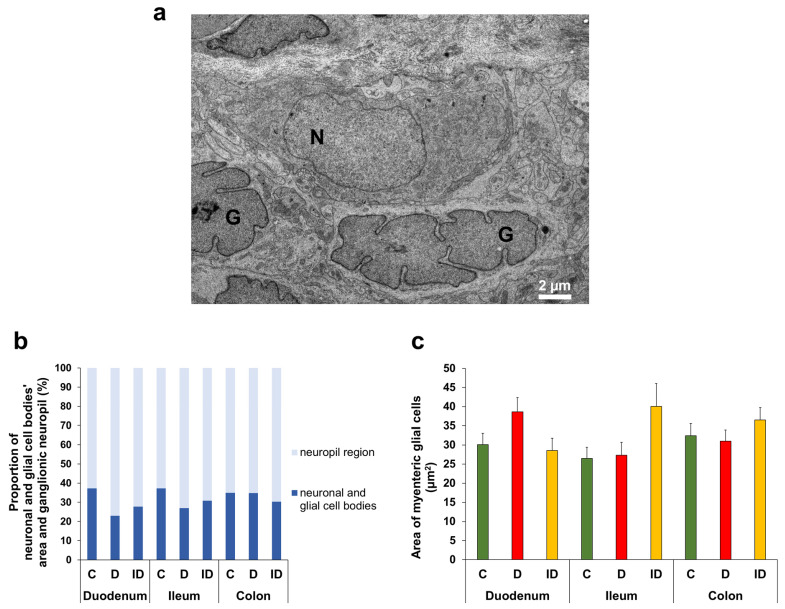
Representative electron micrograph of a myenteric ganglion region from the colon of an insulin-treated diabetic rat of the chronic experiment (**a**). G—nucleus of enteric glial cell; N—nucleus of enteric neuron. Proportion of the area of neuronal and glial cell bodies and neuropil region in the myenteric ganglia (**b**). This proportion was 35–37% in all gut segments of control rats, while it decreased in small intestinal segments of diabetics. Area of myenteric glial cells from different gut segments and conditions (**c**). Glial cell body area demonstrated a slight but not significant increase in the duodenum of diabetic rats relative to controls. Kruskal–Wallis test with Dunn’s multiple comparisons test. Data were expressed as means ± SEMs; *n* = 7–10 myenteric ganglia/segment/experimental group from 6 to 7 animals (**b**) and *n* = 20–30 myenteric glial cells/segment/experimental group from 6 to 7 animals (**c**). C—controls, D—diabetics, and ID—insulin-treated diabetics.

Electron microscopic morphometry of myenteric ganglia elements (Figure 10a) has shown regional changes in the proportion of neuronal and glial cell bodies’ area per total ganglionic area. This proportion was 35–37% in all gut segments of control rats, while it was only 23% in the duodenum, 27% in the ileum and 35% in the colon of diabetic animals (23.05 ± 4.18%; 26.97 ± 5.40%; 34.81 ± 6.02%, respectively) (Figure 10b).

The average area of myenteric glial cells was also measured in different gut segments and experimental groups. In the duodenum, the glial cell body area demonstrated a slight but not significant increase in diabetic rats relative to controls (38.66 ± 3.69 vs. 30.09 ± 2.99 µm^2^), while it did not change in the ileum and colon of diabetic groups (Figure 10c).

### 3.7. Tissue Level of GFAP in Intestinal Homogenates

**Figure 11:**
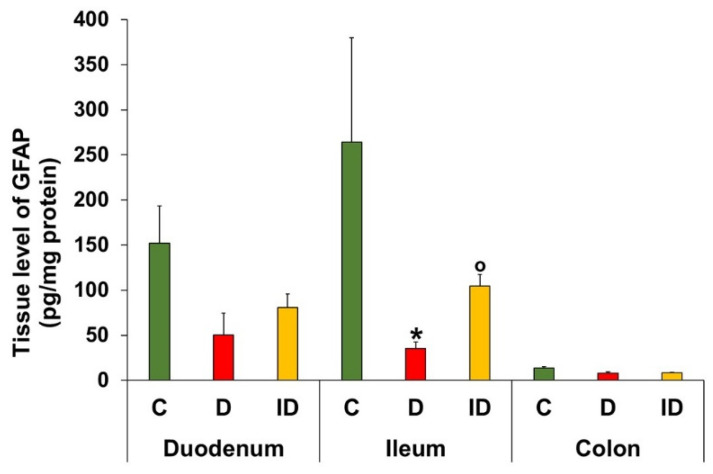
Tissue level of glial fibrillary acidic protein (GFAP) in intestinal homogenates from different gut segments of control (C), diabetic (D) and insulin-treated diabetic (ID) rats of the chronic experiment. Higher GFAP levels were observed in the small intestine than the colon of controls with a decrease in the diabetics. * *p* < 0.05 (relative to controls); ^o^ *p* < 0.05 (between diabetics and insulin-treated diabetics); Kruskal–Wallis test with Dunn’s multiple comparisons test. Data are expressed as means ± SEMs; *n* = 4–6 animals/segment/experimental group.

GFAP levels of myenteric plexus/intestinal smooth muscle homogenates were lowest in the colon of controls (13.51 ± 1.97 pg/mg protein) and remained unchanged in diabetic groups (Figure 11). In the small intestine, higher GFAP levels were observed in the controls, with a decrease in the diabetic group (duodenum: 50.47 ± 24.25 vs. 151.90 ± 41.39 pg/mg protein; ileum: 35.61 ± 6.76 vs. 264.06 ± 115.64 pg/mg protein, *p* < 0.05) (Figure 11). Insulin treatment partially prevented the observed changes in ileal GFAP levels.

## 4. Discussion

Our present study provides the first comprehensive view about Sox10-expressing EGCs in both submucosal and myenteric plexuses and the entire gut wall of different intestinal segments in healthy and type 1 diabetic rats.

Glia and enteric neurons create a close morpho-functional relationship; therefore, it is important to investigate them together. Changes in the glia/neuron ratio could originate from glial and/or neuronal alterations. However, an unchanged ratio could mean either unchanged glial and neuronal densities or parallel and equal changes in both. Moreover, the duration of hyperglycaemia could fundamentally influence these parameters.

In the submucosal plexus, the glial density in the duodenum remained unchanged regardless of the length of hyperglycaemic insults, which suggests a well-balanced duodenal environment [1]. However, in the distal segments, even acute hyperglycaemia resulted in glial changes, supporting the higher vulnerability of the distal gut to diabetic damage [1]. A more favourable microbial composition, redox state and prooxidant/antioxidant milieu of duodenum relative to distal gut were also highlighted in other studies [1,29,30]. While glial density decreased in both the ileum and colon of acute hyperglycaemic animals, it increased in the same segments of chronic ones. It is imaginable that the time-dependent diabetic changes in submucosal glial cell density may reflect a glial loss as a first shock of hyperglycaemic injury, while an increasing glial number may help to maintain the function of submucosal neurons later. An enhanced submucosal glial network and increased expression of glial-derived factors were also observed in colon of rats with irritable bowel syndrome [31].

The myenteric EGCs were also affected by acute hyperglycaemia in the large intestine; however, their number was increased in contrast to the submucosal counterparts. These findings point to two important conclusions: colonic EGCs have the first reaction to hyperglycaemia, and the myenteric and submucosal enteric plexuses respond differently to diabetic damage.

The submucosal glia/neuron ratio was below or close to 1.0 in control rats, meaning the number of glial cells is less or similar to neurons in submucosal ganglia. However, in the myenteric plexus of controls, the number of myenteric glia exceeds neuronal numbers depending on the gut segment. Contradictory or varied data of glia/neuron ratio are available in the enteric or central nervous system of different species, age, sex, intestinal segments or brain regions [23,32,33,34,35]. As it has been referred above, different processes could take place in the background of similar hyperglycaemia-induced alterations of the glia/neuron ratio. For example, in the colonic myenteric ganglia, both acute and chronic hyperglycaemia resulted in an increased glia/neuron ratio. However, it was caused by an enhanced glial number in the acute experiment and a reduced neuronal number in the chronic hyperglycaemic rats.

In our acute experiment, altered glial density of myenteric ganglia was not accompanied by neuronal changes, suggesting that myenteric EGCs are more sensitive to hyperglycaemia than neurons here. Long-lasting hyperglycaemia has already provoked more extensive quantitative changes in the myenteric glial and neuronal populations. Although the total number of myenteric EGCs was markedly decreased in both the proximal and distal parts of the small intestine, the density of gold particles labelling Sox10 was increased only in the glial nuclei of the diabetic duodenum. Total neuronal density was significantly decreased in the ileum and colon but not in the myenteric ganglia of diabetic duodenum. These neuronal alterations are in line with our former observations in the same chronic hyperglycaemic model [27]. Increased Sox10 expression may lead to the upregulation of glial-derived neurotrophic factors [36] and may contribute to neuronal survival in diabetes [37] in the myenteric ganglia of the duodenum.

In the present study, low but regionally distinct amounts of Sox10-HuC/HuD double-labelled cells were observed in myenteric ganglia, and a few cells were detected in submucosal ganglia. The presence of Sox10-HuC/HuD-IR cells in the myenteric but not the submucosal ganglia and their decrease have been also demonstrated in mice during adolescence [38]. The cells that co-express Sox10 and HuC/HuD markers could have neurogenic or gliogenic potential [38]. Enteric neural crest cells initially express Sox10, but as they differentiate into enteric neurons, they begin to express pan-neuronal markers [38,39]. The size of Sox10-HuC/HuD-IR cell bodies was similar to the HuC/HuD-positive neuronal soma size; therefore, these cells could be neurons that may be in a transitional state. It has been demonstrated that neural crest stem cells persist in the adult gut and have restricted potential to form different neuronal subtypes [40].

Our data indicate that both acute and chronic hyperglycaemia affect the proportion of Sox10-HuC/HuD co-expressing cells. Interestingly, long-lasting hyperglycaemia resulted in a 10-times increase in these cells in duodenal myenteric ganglia. Alterations in the intestinal microenvironment or the accumulation of appropriate signals could result in a neurogenic–gliogenic switch of Sox10-positive progenitors [8]. However, the characterisation of Sox10-HuC/HuD co-expressing cells and their possible role in the unchanged number of total myenteric neurons in the duodenum of diabetic rats [27] need further investigation. As a novel finding of the present study, triple-labelling immunofluorescence identified that 90% of Sox10-HuC/HuD co-expressing cells were immunoreactive for nNOS in the colon of controls, while it was nearly 50% in the myenteric ganglia of the duodenum and significantly less in the ileum of controls. Jin et al. [41] proved that upregulation of endogenous nNOS promotes neuronal fate commitment of neuronal stem cells or progenitors through the inhibition of histone deacetylase 2.

As we demonstrated, EGCs were present in both layers of intestinal smooth muscle [9], with smaller numbers in the outer longitudinal layer [15,42]. Several neurotransmitters released by motoneurons stimulate EGCs between the smooth muscle cells and vice versa; glial signals could modulate both smooth muscle cells and motoneurons. Therefore, intramuscular EGCs could stimulate gut motility [43]. According to our results, acute hyperglycaemia already affected intramuscular glia in the large intestine. An acute increase of the density of colonic intramuscular EGCs was followed by a robust decrease in the chronic phase. The loss of EGCs in the gut musculature attenuates smooth muscle motor function [44], which could contribute to prolonged transit times characteristic to diabetes [45].

To provide a more complete view of intestinal glial populations, Sox10 immunoreactivity of mucosal EGCs was also investigated in this study. Their location was close to the intestinal epithelial cells, in agreement with others demonstrating the network of GFAP-positive glial processes [46] and intense S100ß labelling around the crypts [47]. Electron microscopic studies revealed a small distance between the basal lamina of enterocytes and EGCs associated with mucosal nerve fibres [47], suggesting that enteroglia act through paracrine signalling in intestinal barrier functions [48]. Although mucosal EGCs were not quantified in our fluorescent study, their diabetic damage has been shown by other experiments in the mouse small intestine [49]. Significant alterations of the mucosal glial network were also observed in inflammatory bowel disease [50], colonic tumours [47] and gut microbial alterations [51]. Changes in glial homeostasis in the mucosa may disrupt crucial connections of glia with nerve fibres and enteroendocrine and immune cells, contributing to impairments of gastrointestinal functions [23].

GFAP expression originating from myenteric and muscular EGCs of intestinal homogenates varied regionally, with the highest levels in the duodenum and ileum. Furthermore, diabetes resulted in decreased GFAP levels in the small intestine only. These observations are completely in line with the decreased diabetic density of Sox10-IR glia in myenteric ganglia of the duodenum and ileum and unchanged density in the large intestine in our present findings. Similarly, a diabetic decrease in GFAP protein levels was observed in the serum and different brain regions, such as hippocampus, cerebellum, cerebral cortex and brainstem [52,53,54]. However, it should be noted that the diabetic decrease in GFAP levels was not accompanied by altered astrocyte numbers in the brain [52]. Insulin increases the expression of GFAP mRNA and protein in mouse organotypic cultures [55]; therefore, the absence of insulin production may be linked to reduced GFAP levels in diabetics. However, contradictory results have also been published depending on the gut segment, duration of hyperglycaemia and type of diabetes [37,49,56].

## 5. Conclusions

In summary, quantitative mapping of different types of EGCs enmeshing the gut wall was completed in the present study. Ganglionic, intramuscular and mucosal densities of EGCs reveal intestinal regional differences even in a control state. Moreover, the duration of hyperglycaemia has time-dependent effects on glial density in different gut segments. An increased or reduced number of EGCs in diabetes itself has a great impact on the functions of submucosal and myenteric neurons and contributes to diabetic damage of gut motility, demonstrated earlier in our same diabetic model [27]. However, the underlying mechanisms of glial response to hyperglycaemia are multifarious. Redoxosome activation leading to glial oxidative damage, reactive gliosis promoting cytokine secretion, and enhanced or decreased release of neurotrophic factors are all involved in diabetic neuropathy [57,58,59] and need further study.

## Figures and Tables

**Table 1:** Primary and secondary antibodies used in the fluorescent experiments.

**Primary Antibody**	**Host**	**Final** **Dilution**	**Product Code**	**Company**
anti-Sox10 monoclonal	mouse	1:100	sc-365692	Santa Cruz Biotechnology, Dallas, TX, USA
anti-HuC/HuD monoclonal	rabbit	1:200	ab184267	Abcam, Cambridge, UK
anti-nNOS polyclonal	guinea pig	1:500	432005	Synaptic Systems GmbH, Goettingen, Germany
**Secondary Antibody**	**Host**	**Final** **Dilution**	**Product Code**	**Company**
anti-mouse Cy^TM^3	goat	1:200	115-165-003	Jackson ImmunoResearch Laboratories, West Grove, PA, USA
anti-rabbit Alexa Fluor^®^ 488	goat	1:200	A11008	Invitrogen, Thermo Fisher Scientific, Waltham, MA, USA
anti-guinea pig Cy5^®^	goat	1:200	ab102372	Abcam, Cambridge, UK

**Table 2:** Body weight and blood glucose levels of the animals in the acute and chronic models of type 1 diabetes.

**Acute Experiment**	**Body Weight (g)**		**Blood Glucose Concentration (mmol/L)**	
	Initial	Final	Initial	Final (Average)
Controls (*n* = 10)	235.0 ± 5.63	303.8 ± 8.56 ****	6.08 ± 0.36	6.52 ± 0.10
Diabetics (*n* = 11)	222.1 ± 5.16	261.1 ± 4.44 ****^, oo^	5.45 ± 0.26	29.85 ± 0.83 ****^, oooo^
**Chronic Experiment**	**Body Weight (g)**		**Blood Glucose Concentration (mmol/L)**	
	Initial	Final	Initial	Final (Average)
Controls (*n* = 11)	216.7 ± 5.06	475.1 ± 19.03 ****	5.85 ± 0.37	5.99 ± 0.13
Diabetics (*n* = 12)	229.8 ± 11.04	320.7 ± 18.36	5.75 ± 0.34	30.04 ± 0.90 ****^, oooo^
Insulin-treated diabetics (*n* = 12)	219.3 ± 5.65	458.3 ± 15.53 ****	5.81 ± 0.38	13.44 ± 1.15 **^, o^

Data are expressed as mean ± SEM; ** *p* < 0.01 and **** *p* < 0.0001 vs. initial; ^o^ *p* < 0.05, ^oo^ *p* < 0.01, and ^oooo^ *p* < 0.0001 vs. final controls.

## Data Availability

The raw data supporting the conclusions of this article will be made available by the authors on request.

## Abbreviations

The following abbreviations are used in this manuscript:

EGCs	Enteric glial cells
GFAP	Glial fibrillary acidic protein
IR	Immunoreactive
nNOS	Neuronal nitric oxide synthase
STZ	Streptozotocin
